# Addition of lime juice and NaCl to minced seafood may stimulate the expression of *Listeria monocytogenes* virulence, adhesion, and stress response genes

**DOI:** 10.1002/fsn3.4064

**Published:** 2024-02-28

**Authors:** Hedayat Hosseini, Esmail Abdollahzadeh, Zahra Pilevar

**Affiliations:** ^1^ Department of Food Science and Technology, National Nutrition and Food Technology Research Institute, Faculty of Nutrition Sciences and Food Technoloy Shahid Beheshti University of Medical Sciences Tehran Iran; ^2^ International Sturgeon Research Institute, Iranian Fisheries Science Research Institute, Agricultural Research Education and Extension Organization (AREEO) Rasht Iran; ^3^ School of Health Arak University of Medical Sciences Arak Iran

**Keywords:** lime juice, *Listeria monocytogenes*, minced seafood, NaCl, virulence and adhesion genes

## Abstract

*Listeria monocytogenes* is a ubiquitous opportunistic bacterium responsible for deadly listeriosis outbreaks. This pathogen has been recognized as a significant food‐borne pathogen in seafood products. The present study aimed to investigate the transcript levels of virulence, adhesion, and stress response genes of *L*. *monocytogenes* upon exposure to sublethal levels of lime juice and NaCl in shrimp matrix. For this purpose, minced and broth shrimp samples (control, 2% NaCl, 5% NaCl, 25 μL/mL lime, and 50 μL/mL lime, as well as 2% NaCl+25 μL/mL lime) were inoculated with approximately 10^7^ CFU/g or ml of *L*. *monocytogenes*, and subsequently, the samples were stored at 12°C or 37°C. For the minced samples, the transcription of one stress‐related (*sigB*), two adhesion (*imo1634* and *imo1847*), and four virulence (*hly*, *prf*, *intA*, and *plc*) genes was assessed by RT‐qPCR after different storage times (0 and 48 h). Results showed that the transcript levels of *sigB*, *imo1847*, and *imo1634* genes increased with increasing storage temperatures of shrimp broth (12°C to 37°C). At the beginning, the transcription of the studied genes decreased in all treatments of minced shrimp; however, after 48 h of storage at 12°C, the transcript levels of *hly*, *prf*, *imo1847*, *imo1634*, and *intA* genes were significantly upregulated up to 0.5–9 log_2_ fold‐change in all treatments compared to the control group (*p* < .05). These results highlight that the survived *L*. *monocytogenes* after exposure to moderate salt content or lime juice could represent enhanced virulence and adhesion capabilities, posing a significant public health risk.

## Graphical abstract

Temperature plays a crucial role in the expression of adhesion and stress genes Effect of lime juice and NaCl on expression of stress, adhesion, and virulence genes

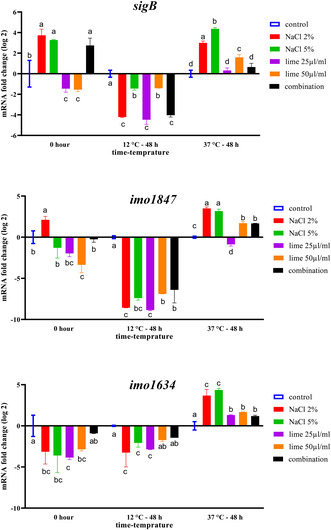

## 1 INTRODUCTION

*Listeria monocytogenes* is an opportunistic ubiquitous Gram‐positive psychrotrophic food‐borne bacterium that is the only pathogenic species for humans among the 18 known species of the genus *Listeria* (Benlloch‐Tinoco et al., 2014; Olaimat et al., 2018). This food‐borne pathogen, as the most common cause of Class I recalls, has a low incidence rate (0.1–11.3 cases per million) with a high mortality rate of 20%–30% (Abdollahzadeh, Mahmoodzadeh Hosseini, & Imani Fooladi, 2018; Abdollahzadeh, Ojagh, et al., 2018; De Souza et al., 2008; Ivanek et al., 2005).

*Listeria monocytogenes* can survive in a wide range of harsh environmental conditions, such as high salt (20%), low temperature (frozen products), and pH values from 4.3 to 9.6 (Olaimat et al., 2018). *Listeria monocytogenes* can survive and even proliferate in adverse environmental conditions. The virulence activity of this bacterium can be affected by exposure to intrinsic and extrinsic stress factors (Bahrami et al., 2020; Pilevar et al., 2020).

Raw and processed seafood are significant vehicles for the contamination of *L*. *monocytogenes* (Hosseini et al., 2021; Zhao et al., 2020). Shrimp, as the most economically important internationally traded seafood, has a favorable condition for the growth of microorganisms during handling and processing (FAO, 2020). As reported by the FDA, *L*. *monocytogenes* is one of the most common causes of seafood product recalls. For example, Norpac Fisheries Export recalled all fresh shrimp distributed in the Hawaiian Islands between May 2020 and June 2020 because of potential contamination with *L*. *monocytogenes*.

Traditionally, NaCl and lime juice, as flavor‐enhancing agents, are added to seafood such as fish, shrimp, crabs, and lobster. Although NaCl, as a distinctive cheap additive, has a vital role in the flavor, texture, and safety of seafood products, it is recommended to reduce the consumption of sodium chloride due to cardiovascular, autoimmune, and kidney diseases caused by this salt (Haase et al., 2019). The concentration of NaCl in foods can be reduced by applying additional hurdles, such as acidification or lowering pH with natural preservatives. Lime juice, with a low pH of 2–3, can be applied to seafood products as an antioxidative and antimicrobial agent, which needs to be studied more (Daundasekara & Rajapaksha, 2021; Lota et al., 2002).

Due to the risk to public health, the transcriptomic responses of food‐borne pathogens in food matrices have attracted significant scientific interest in recent years. Recently, Chen et al. (2021) presented an overview of the transcriptomic responses of foodborne pathogens to the food matrices. In our previous work, the transcript levels of *L*. *monocytogenes* virulence‐associated genes were assessed in the fish medium after exposure to thyme essential oil (EO) as a natural preservative. The results of the study revealed that thyme EO could increase the virulence potential of *L*. *monocytogenes*. Hence, it is critical to examine how natural preservatives and food matrices may alter *L*. *monocytogenes* behaviors, especially survival, virulence, biofilm, and adhesive properties. To the best of our knowledge, there are no reported data about the anti‐Listeria activities of lime juice and salt content, as well as the transcript levels of virulence and adhesion of *L*. *monocytogenes* in shrimp matrix.

Therefore, this study aimed to investigate the transcript levels of virulence, adhesion, and stress response genes of *L*. *monocytogenes* upon exposure to sublethal levels of lime juice and NaCl in the shrimp matrix.

## 2 METHODS

### 2.1 Bacterial strains

One *L*. *monocytogenes* strain was used in this work. This strain was previously isolated from seafood samples (Abdollahzadeh et al., 2016) and kindly sequenced and stored by Dr. Leclercq in the Institut Pasteur with 17/01036 number.

The bacterium was stored in a stock culture (TSB+30% glycerol at −20°C) and was revived in BHI broth (brain heart infusion) at 37°C for 28 h. After two consecutive transfers and culture in BHI, the bacterium was inoculated into the broth and minced shrimp matrices. The bacterial cells were separated from the culture medium by triple centrifugation at 6000 rpm for 5 min. The pelleted cells were washed 3 times with physiological serum. The bacterial cell count was determined by the optical density method at 600 nm. The optical density of 0.1–0.08 was equal to 1 × 10^8^ CFU/mL (Abdollahzadeh, Mahmoodzadeh Hosseini, & Imani Fooladi, 2018; Abdollahzadeh, Ojagh, et al., 2018). The population of bacteria was checked with the pour plate method at 37°C for 24–48 h.

### 2.2 Preparation of shrimp treatments

The shrimp samples were purchased from a local market in Tehran. The fresh shrimp samples were peeled, deveined, and minced. The minced shrimp was mixed with distilled water (2:1 w/v; water: minced shrimp) and boiled for 20 min. Then, the suspension was filtered with sterile gauze. For preparing NaCl treatments, after the addition of 2% or 5% NaCl to the broth (1 g NaCl in 50 mL of shrimp broth or 1.5 g NaCl in 30 mL of shrimp broth; pH = 7.03), the suspensions were sterilized at 121°C for 15 min and kept in a refrigerator. To prepare lime treatments, the shrimp broth was sterilized, and then appropriate volumes of lime juice (25 and 50 μL/mL) were added aseptically to the broth. A combination of NaCl and lime juice treatments (2% and 25 μL/mL) was also prepared. The minced shrimp treatments were prepared as treatments for the shrimp broth, with minor modifications. After preparing the treatments, an appropriate volume of the *L*. *monocytogenes* inoculum was added to shrimp matrices to adjust the final bacterial concentration to approximately 7 log CFU/g or ml (minced or broth treatments). Until RNA extraction intervals (0 and 48 h), all inoculated broth treatments were incubated at 12°C, considered as the abuse temperature for cold storage, and 37°C, as the optimum growth temperature for the bacterium. The minced shrimp samples were only kept at 12°C. Three replicates of each treatment were used for the gene expression assay.

### 2.3 Determination of minimum inhibitory concentration (MIC) in culture medium and shrimp broth

As viable cells of bacteria are needed for assessing gene expression, sub‐MIC concentrations of lime or NaCl were employed in the study. For this purpose, the serial macro‐dilution method was used, with different levels of lime or NaCl added to the TSB or shrimp broth. The negative and positive controls were prepared. After inoculating 10^7^ CFU/mL *L*. *monocytogenes* into the serial dilutions, the TSB or shrimp broth media were incubated at 37°C. The MIC was determined as the lowest concentration showing no turbidity after 24 h (Pilevar et al., 2020). Due to the high turbidity of shrimp broth, the MIC was determined by the pour plate method after the incubation. The MIC was the lowest concentration of lime at which the bacterial population was the same as 0 h (10^7^ CFU/mL). To determine MBC, 100 μL of dilutions showing no bacterial growth were cultured on TSB agar and incubated at 37°C for 24 h. The lowest concentration of lime juice exhibiting no colony growth was determined as MBC (Pilevar et al., 2020).

### 2.4 Enumeration of *L*. *monocytogenes*

For microbial analysis, 1 mL (or 1 g) of shrimp broth (or minced shrimp) samples were homogenized with 9 mL of physiological serum. Serial dilutions were made, and counts of the bacterium were determined by the pour plate method in PALCAM *Listeria* selective medium. The plates were incubated at 37°C for 24–48 h.

### 2.5 Gene expression assay

The transcript levels of virulence (*hly*, *prf*, *intA*, and *plc*; Table 1), adhesion (*imo1634* and *imo1847*; Table 1), and stress response (*sigB*; Table 1) genes were assessed in the minced shrimp samples at 12°C. For the shrimp broth treatments, the expression of adhesion and stress response genes was also investigated after 0 h or 48 h of storage at 12°C or 37°C. Here, the sampling at 0 h was performed approximately 5 min after the bacterial inoculation.

**TABLE 1:** Primers used in this study.

Gene	Forward primer (5′‐3′)	Reverse primer (5′‐3′)
*16s*	GATGCATAGCCGACCTGAGA	CTCCGTCAGACTTTCGTCCA
*hly*	TACATTAGTGGAAAGATGG	ACATTCAAGCTATTATTTACA
*Plc*	CTAGAAGCAGGAATACGGTACA	ATTGAGTAATCGTTTCTAAT
*inlA*	TGTTACAAGAACCTACGGCACCAACAA	TTGGCGCTATATTGGGCATATAAGGTGATG
*lmo1634*	GTTGTTGCCGGCGTTACAC	CGCGATAATTGCTTTGAAAAGA
*lmo1847*	GCGTGGATCCGCATGAAT	GCATCCGCAGCACTTTGAAT
*sigB*	CCAAGAAAATGGCGATCAAGAC	CGTTGCATCATATCTTCTAATAGCT

### 2.6 RNA extraction, purification, and quality control

The total RNA of inoculated *L*. *monocytogenes* cells (>7 log CFU/g or ml) in shrimp samples was extracted according to the manual of the GeneAll Hybrid‐R™ extraction kit (GeneAll Biotechnology Co., Ltd., Korea). Due to the sensitivity of RNA to RNases and degradation, there was no gap between sampling and isolating RNA. Immediately after sampling, RNA extraction was performed according to the instructions of the manufacturer. 200 μL of RNA*later*® solution was mixed with the samples to stabilize the RNA. Next, RiboEx™ (provided in the kit) was added to samples in order to inactivate RNases. The quality and quantity of RNA samples were assessed using the NanoDrop® spectrophotometer (Thermo Fisher Scientific Inc., Waltham, MA, USA).

### 2.7 cDNA synthesis

PrimeScript™ RT reagent kit was employed to synthetize the double‐stranded cDNA from RNA. According to the kit protocol, the reaction mixture (2 μL of 5X PrimeScript Buffer, 0.5 μL of PrimeScript RT Enzyme Mix I, 0.5 μL Oligo dT Primer, 0.5 μL Random 6 mers, 2.5 μL of RNase Free dH_2_O, and 4 μL of RNA) was prepared on ice and then incubated at 37°C for 15 min for reverse transcription. Finally, the reaction was kept at 85°C for 5 s for the inactivation of reverse transcriptase. The synthesized cDNA was stored in the freezer (Pilevar et al., 2020).

### 2.8 Real‐time PCR

The qRT‐PCR assay was conducted in a 25‐μL reaction mixture (12.2 μL water, 2 μL cDNA, 10 μL of 2x q PCR Master Mix Green high Rox, 0.4 μL forward and 0.4 μL reverse primers). The qRT‐PCR condition included a holding stage of 15 min at 95°C, followed by 40 cycles at 95°C for 25 s, at 60°C for 30 s, and 72°C for 30 s. Primer specificity was assessed by a melting curve analysis. After normalization of the Ct values of target genes with the Ct of the housekeeping gene (*16s*), the relative gene expression (fold‐change) was calculated using the 2^−ΔΔCt^ method, and log_2_ (fold‐change) were reported (Ersoy et al., 2021; Pérez‐Baltar et al., 2022). *IGS* and *rpob* was also used as housekeeping genes in our pretests; however, *16s* was employed as the most stable reference gene for this study. At the same time and temperatures, shrimp samples (broth or meat) without preservatives (NaCl or lime) were employed as a reference group (control) for the calculation of mRNA fold change in the treatments (NaCl, lime, and their combination).

### 2.9 Statistical analysis

After checking the normal distribution of data with the Kolmogorove–Smirnov test, the ANOVA test was employed to compare the data on gene expression. Differences among treatments were considered significant at *p* < .05.

## 3 RESULTS AND DISCUSSION

### 3.1 MIC and MBC of lime and NaCl in shrimp broth and the counting of inoculated *L*. *monocytogenes* cells

Lime and lemon juices are highly consumed as popular fruit products that contain high contents of vitamins, phenolic compounds, and antimicrobial agents (Arian et al., 2019). Lime (*Citrus aurantifolia* Swingle) is mainly produced in the southern locations of Iran (e.g., Jahrom and Minab). In the present study, the lime samples were collected from Jahrom. Some variations in the physicochemical characteristics can be observed based on the growth locations. The range of total acidity, pH, total solid (g/100 g sample), and vitamin C concentrations (mg/100 g sample) of lime samples (collected from the various locations of Iran) were reported as 5.8–6.5, 2.4–2.5, 8.8–9.1, and 34.2–37.9, respectively (Ansari & Rezaei, 2008).

The MIC and MBC of lime juice were 125 μL/mL and 375 μL/mL in shrimp broth, respectively. The MIC in TSB broth was 75 μL/mL. A higher MIC value in shrimp broth than that of liquid culture medium (TSB) indicates that shrimp broth is a rich medium to support bacterial growth or protect this pathogen from the effects of antimicrobial agents. In our previous study, the MIC of NaCl was 10% against *L*. *monocytogenes* (Abdollahzadeh et al., 2021).

The bacterial count was performed at 0 and 48 h to confirm the dose inoculated to shrimp broth treatments. The inoculated microbial population at 0 and 48 h of storage (12°C) was 7.22 and 9.04 log CFU/g, respectively (Table 2). During storage at 12°C, bacterial counts in treated groups slightly increased with elapsed time. Therefore, at least 7 log CFU/g was used to extract RNA for gene expression experiments.

**TABLE 2:** The inoculated *Listeria monocytogenes* population in shrimp treatments during 0 and 48 h of storage at 12°C.

Time (h)	Treatment	Population (log CFU/g)
0	All	7.22
48	Control	9.38
48	2% NaCl	8.87
48	5% NaCl	8.32
48	25 μL/g	9.11
48	50 μL/g	9.07
48	Combination (2% NaCl +25 μL/g)	8.89

### 3.2 The effects of NaCl and lime juice on the *L*. *monocytogenes* gene expression in broth and minced shrimp medium

**FIGURE 1:**
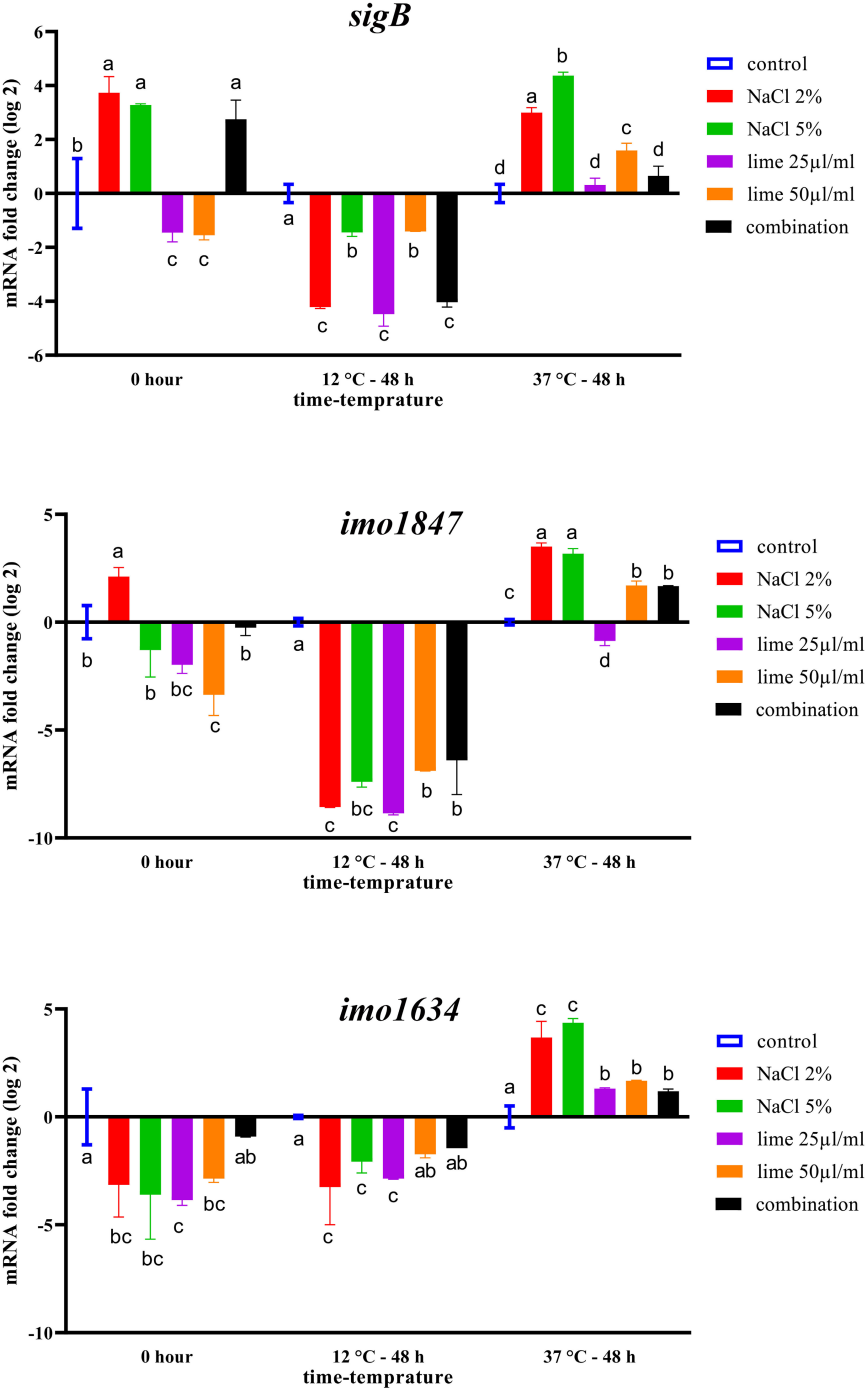
The effects of NaCl, lime, and their combinations on the transcript levels of *imo1847*, *imo1634*, and *sigB* genes in shrimp broth during storage at 12°C and 37°C for 48 h. Significant differences are displayed with various letters (*p* < .05).

Figure 1 displays the effects of NaCl and lime juice on the transcription of *sigB*, *imo1634*, and *imo1847* genes of *L*. *monocytogenes* in shrimp broth. The gene expression was measured in shrimp broth at two‐time intervals (0 and 48 h of storage) and two storage temperatures (12 and 37°C). At 0 h, the expression of adhesion genes (*imo1847* and *imo1634*) decreased in all treatments (except for the *imo1847* gene in 2% NaCl) compared to the control group. However, the transcript level of the *sigB* gene at 0 h was increased in NaCl (2% and 5%) and combination treatments (Figure 1). Moreover, after 48 h of shrimp broth storage at 12°C, the expression of adhesion genes were significantly downregulated compared to the control group (*p* < .05). This downregulation in the *imo1847* gene was more than *imo1634* (Figure 1). Similar results were obtained for *sigB* after 48 h of storage at 12°C. After 48 h of storage at 12°C, the *imo1847* transcript level in all treatments was about 7‐ to 9‐fold change (log2) lower than the control group. As shown in Figure 1, by storing shrimp broth at 37°C, different results were obtained for the adhesion genes. For example, despite the storage temperature at 12°C, the transcription of adhesion and *sigB* genes at 37°C was significantly overexpressed in most treatments compared to the control group (*p* < .05). Therefore, it can be concluded that storage temperature plays a crucial role in the expression of adhesion and stress genes.

**FIGURE 2:**
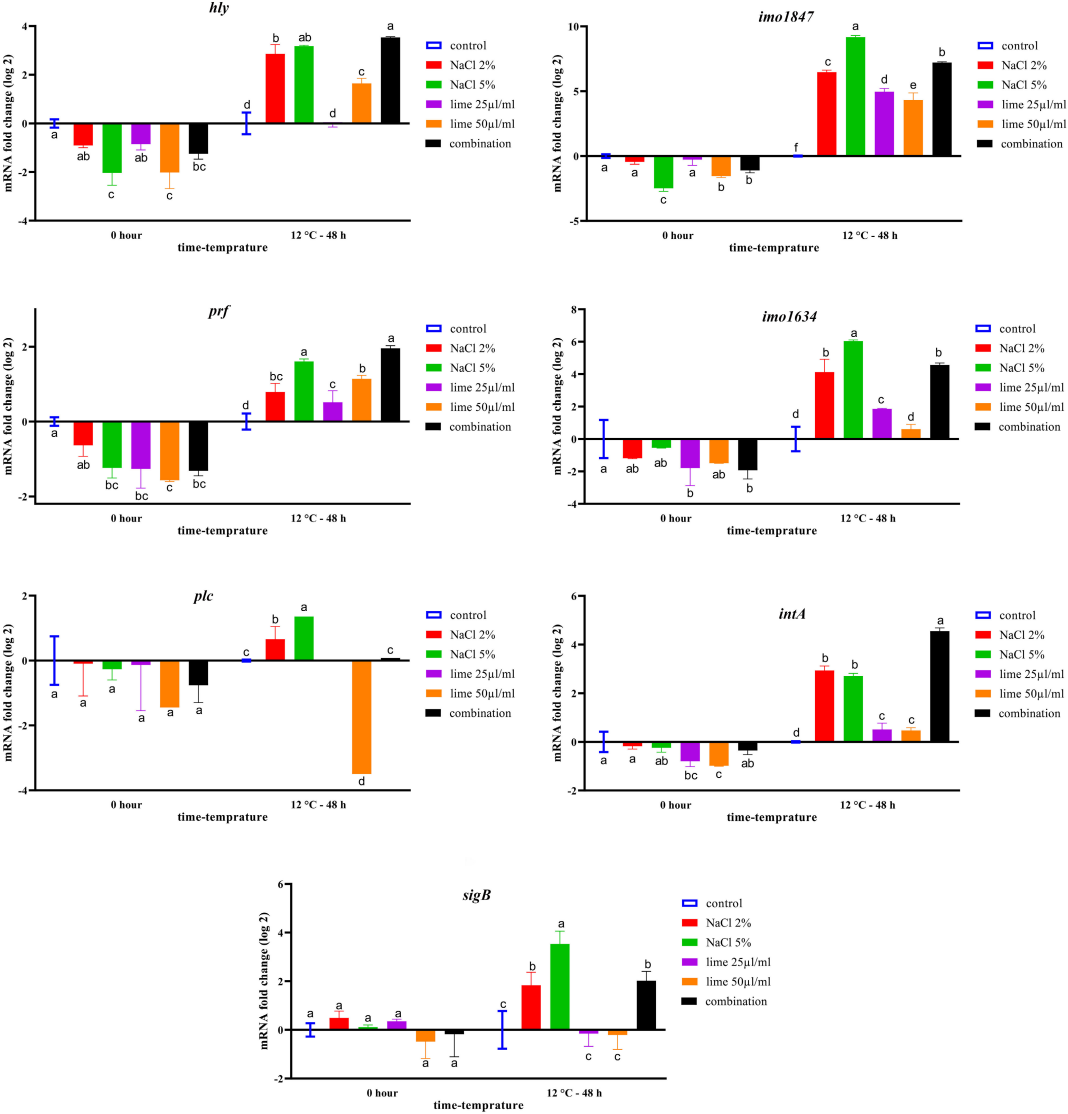
The effects of NaCl, lime, and their combinations on the transcript levels of *imo1847*, *imo1634*, *sigB*, *hly*, *prf*, *plc*, and *intA* genes in minced shrimp medium during storage at 12°C for 48 h. Significant differences are displayed with various letters (*p* < .05).

Figure 2 demonstrates the effects of NaCl, lime juice, and their combination on the expression of adhesion, stress, and virulence genes in minced shrimp meat. Interestingly, at the beginning, the transcription of the studied genes decreased in all treatments; however, the overexpression of the *sigB* gene was observed in some treatments at 0 h. These findings were similar to the expression of *sigB* in shrimp broth.

The use of 50 μL/g lime juice reduced the expression of *intA*, *prf*, and *hly* genes in minced meat at 0 h (*p <* .05; Figure 2); however, the use of NaCl (5%) was more effective in downregulating the imo1847 gene at 0 h. In addition, there was no significant difference between the expression level of the *hly* gene in 5% NaCl and 50 μL/g lime juice treatments at 0 h (*p* > .05).

After 48 h of storage at 12°C, the expression of *hly*, *prf*, *imo1847*, *imo1634*, and *intA* genes significantly increased in all treatments compared with the control treatment (*p* < .05). These results were similar to those found in shrimp broth. On the other hand, after 48 h of storage, the transcription of *plc* and *sigB* genes was relatively downregulated by the lime juice treatment.

After 48 h of storage at 12°C, the combination of NaCl (2%) and lime juice (25 μL/g) raised the transcription of *hly*, *prf*, and *intA* genes. The overexpression of *hly* and *prf* genes in the combination treatment was significantly higher than other treatments (i.e., control, lime juice, and 2% NaCl groups); however, there was no significant difference between the combination group and the 5% NaCl treatment. After 48 h of storage, the 5% NaCl treatment induced higher expression of *sigB*, *imo1847*, *imo1634*, and *plc* genes compared with other treatments. The 5% NaCl and the combination treatments, in general, induced the highest transcription of almost all of the assessed genes at 12°C after 48 h.

The transcriptional response of virulence and adhesion genes is not a stable characteristic of pathogenic bacteria. Environmental factors, physicochemical conditions, and host characteristics may influence the gene expression of food‐borne pathogens. Limited information is available on the effect of food matrix and food preservatives on the expression of *L*. *monocytogenes* genes. In the present work, the transcript levels of sigB, imo1847, and imo1634 genes increased with increasing storage temperature (12°C to 37°C; Figure 1). These results agree with those reported by Duodu et al. (2010) and Hadjilouka et al. (2016), who reported that the expression of virulence‐ or adhesion‐related genes in *L*. *monocytogenes* increases at higher temperatures. Pilevar et al. (2020) investigated the transcript levels of *hly*, *prfA*, *inlA*, and *inlB* genes after exposure of *L*. *monocytogenes* to the subMIC concentrations of *Zataria multiflora* Boiss essential oil in fish broth and minced fish mediums. They found that adding essential oil to the broth and minced rainbow trout fish can significantly upregulate the virulence genes.

Several studies have been conducted to investigate the effect of NaCl and acidic conditions on the gene regulation of *L*. *monocytogenes* in the food matrix. For example, Liu et al. (2014) examined the effect of NaCl on the *L*. *monocytogenes* genes involved in cell wall synthesis, cell division, and NADPH production in refrigerated vacuum‐packaged ham. Their results showed that after 1 month of ham storage, ftsX was downregulated with 2.35% of NaCl; however, after 3 months, the transcription of *ftsX*, *murZ*, and *gnd* genes upregulated 35‐, 7‐fold, and 4‐fold, respectively. Alía et al. (2019) investigated the effect of a_w_, temperature, and NaCl on the *plcA*, *hly*, *sigB*, and *iap* genes in a dry‐cured ham. They reported that the salt levels decreased the expression of the virulence gene depending on the a_w_. However, the sigB expression was stimulated at moderate NaCl and water activity conditions (at 15°C). This overexpression of the sigma factor at 3% NaCl was also demonstrated in Kazmierczak et al. (2003) and Olesen et al. (2010). These findings were similar to the results of the current study on minced shrimp. It can be concluded that in stressful conditions, the expression of the *sigB* gene may be activated to increase *L*. *monocytogenes* survival. Additionally, *sigB* also contributes to the transcript level of the *prfA* gene, which is well established as an essential transcriptional regulator of virulence genes (i.e., *hly* and *plcA*) (Lucas et al., 2021; Ollinger et al., 2009). Olesen et al. (2009) investigated the effects of acidic (pH 5.5) and NaCl (4.5%) stress on the virulence potential of two strains of *L*. *monocytogenes* in a culture medium. They confirmed that long‐term acidic and NaCl stress can increase *L*. *monocytogenes* virulence, which was in agreement with the current study in minced shrimp. Regarding these studies, in response to NaCl and acidic conditions, it can be concluded that mixed responses to gene regulation have been observed for this pathogen.

The high variation in the findings of research can be related to temperature, food conditions, and strain variations (Behari & Youngman, 1998). As suggested in research, the exposure of bacteria to stressful conditions can resist the pathogens not only to those stress factors but might also enhance cross‐protection against other stresses such as preservatives and processing environments (Pilevar et al., 2020; Walsh et al., 2001). Based on the results of the current study, although using low concentrations of NaCl and lime juice or their combination in minced shrimp can downregulate the virulence and adhesion genes at 0 h, these preservatives act as stress factors and increase the severity of the *L*. *monocytogenes* in seafood with elapsed time. As the regulation of genes in seafood and the human body might be different, in vivo studies are needed to approve this hypothesis.

## 4 CONCLUSION

For the first time, the transcription levels of the critical virulence and adhesion genes of *L*. *monocytogenes* were evaluated following exposure to NaCl, lime juice, and their combination in broth and minced shrimp matrices. The regulation of virulence and adhesion genes was affected by storage time and temperature, as well as the concentration of NaCl and lime juice. In all treatments of minced shrimp taken together, the transcript level of the studied genes decreased at the beginning. However, after 48 h of storage at 12°C, the transcription of *hly*, *prf*, *imo1847*, *imo1634*, and *intA* genes significantly increased to 0.5–9 log2 (fold‐change) in all treatments compared to the control group. Due to the severe pathogenicity of *L*. *monocytogenes*, more research needs to be conducted on virulence and adhesion genes in both food matrices and in vivo condition.

## AUTHOR CONTRIBUTIONS

**Hedayat Hosseini:** Formal analysis (supporting); funding acquisition (lead); project administration (lead); resources (lead); supervision (supporting). **Esmail Abdollahzadeh:** Data curation (lead); formal analysis (lead); investigation (equal); methodology (lead); software (lead); visualization (lead); writing – original draft (lead); writing – review and editing (lead). **zahra pilevar:** Formal analysis (equal); investigation (equal); methodology (supporting); writing – original draft (supporting); writing – review and editing (supporting).

## CONFLICT OF INTEREST STATEMENT

The authors have no conflicts of interest to declare.

## Data Availability

All of the data generated or analyzed during this study are included in this paper. In case more data is needed for specific purposes, it is available from the corresponding author on request.
